# Nutritional Density and Its Impact on Biological Age: Insights From Large Cohort Studies

**DOI:** 10.1093/geroni/igaf122.3982

**Published:** 2025-12-31

**Authors:** Eleonora Porcu, Charlotte Debras, Shu Wang, Li-Tang Tsai, Alberto Conde Freniche, Philipp Gut, Sébastien Herzig

**Affiliations:** Nestle Institute of Health Science, Lausanne, Vaud, Switzerland; Nestle Institute of Health Science, Lausanne, Vaud, Switzerland; Nestle Institute of Health Science, Lausanne, Vaud, Switzerland; Nestle Institute of Health Science, Lausanne, Vaud, Switzerland; Nestle Institute of Health Science, Lausanne, Vaud, Switzerland; Nestle Institute of Health Science, Lausanne, Vaud, Switzerland; Nestle Institute of Health Science, Lausanne, Vaud, Switzerland

## Abstract

Relationships between nutrition and aging represent a critical area of research, as dietary choices can influence health and disease risk. In this study, we used Clinical PhenoAge (PA) as an indicator of health status reflecting biological processes of aging better than chronological age. We calculated biological age advancement (BAA) in two populations (UKBiobank, N = 103,344 and NHANES 2015-2018, N = 4,264) as the difference between PA and chronological age and explored the association with specific diet quality scores. To this end we used nutrition density (measured using NRF9.3 score, a method evaluating adequate intakes of 12 nutrients) as well as individual nutrient intake. After adjusting for relevant anthropometric, sociodemographic and health-related covariates, the linear regression analyses revealed that a 50-point increase of NRF9.3 is associated with lower BAA in UKBiobank (β=-0.093[95% CI: -0.103,-0.083], P < 0.001) and in NHANES (β=-0.152[-0.192,-0.112], P< 0.001). Given that BMI is often influenced by dietary choices and could be associated with PA, we aimed to investigate whether the association between NRF9.3 and BAA could be mediated by BMI. Mediation analysis indicated that a modest part of the association was mediated by BMI (19%[16%-22%] in UKBiobank, and 12%[6%-19%] in NHANES, P < 0.001). Furthermore, we identified specific nutrients associated with lower BAA, including fiber (β_UKBiobank=-0.11[-0.13,-0.09], β_NHANES=-0.44 [-0.59,-0.30], P< 0.001) and magnesium (β_UKBiobank=-0.11[-0.13,-0.09], β_NHANES=-0.46[-0.60,-0.31],P< 0.001). Conversely, nutrients associated with higher BAA included added sugars (β_UKBiobank=0.15[0.13,0.17], β_NHANES=0.35[0.21,0.50], P< 0.001). Overall, these findings suggest the relevant role of diet in biological aging, highlighting the potential for targeted nutritional interventions promoting healthier aging, ultimately leading to healthy longevity.

